# Redundancy and Synergy of an Entangling Cloner in Continuous-Variable Quantum Communication

**DOI:** 10.3390/e24101501

**Published:** 2022-10-21

**Authors:** Vladyslav C. Usenko

**Affiliations:** 1 Department of Optics, Palacky University, 17. Listopadu 12, 77900 Olomouc, Czech Republic; usenko@optics.upol.cz; 2 Bogolyubov Institute for Theoretical Physics of National Academy of Sciences of Ukraine, Metrolohichna St. 14-b, 03680 Kyiv, Ukraine

**Keywords:** quantum communication, continuous variables, quantum entanglement, quantum key distribution, entangling cloner

## Abstract

We address minimization of information leakage from continuous-variable quantum channels. It is known, that regime of minimum leakage can be accessible for the modulated signal states with variance equivalent to a shot noise, i.e., vacuum fluctuations, in the case of collective attacks. Here we derive the same condition for the individual attacks and analytically study the properties of the mutual information quantities in and out of this regime. We show that in such regime a joint measurement on the modes of a two-mode entangling cloner, being the optimal individual eavesdropping attack in a noisy Gaussian channel, is no more effective that independent measurements on the modes. Varying variance of the signal out of this regime, we observe the nontrivial statistical effects of either redundancy or synergy between the measurements of two modes of the entangling cloner. The result reveals the non-optimality of entangling cloner individual attack for sub-shot-noise modulated signals. Considering the communication between the cloner modes, we show the advantage of knowing the residual noise after its interaction with the cloner and extend the result to a two-cloner scheme.

## 1. Introduction

Ability to transmit information in an advanced way, impossible within the classical realm, is an important feature of quantum states, studied by quantum communication. However, during propagation through a quantum channel the states interact with the environment and part of the information becomes shared with it. Such information leakage is essential when security of quantum key distribution (QKD) [1] is considered. Indeed, as follows from the Csiszár-Körner theorem [2], secure key can be distilled from the partially correlated data when the mutual information between the trusted parties exceeds the upper bound on the information which is leaking to the untrusted channel, i.e., is available to a potential eavesdropper. Assessment of this quantity depends on the presumable effectiveness of measurement, which an eavesdropper is capable of. In the most feasible case of individual measurements on the leaking signal, which do not require efficient quantum memories, needed for more advanced collective measurements, the upper bound is given by the Shannon classical information [3]. It was shown for continuous-variable (CV) [4] squeezed signal states that controllable modulation allows reduction of information leakage to an untrusted noisy environment [5] and its complete cancellation once the environment is purely lossy [6] even when an eavesdropper is capable of collective attacks. In this paper we address the minimization of information leakage in the individual attacks case, derive the respective condition and study the properties of the mutual information in the regime of minimum information leakage as well as out of this regime. In the case of individual attacks, a generally noisy environment can be optimally modeled as an entangling quantum cloner [7]. While such an eavesdropping attack can reach the bounds set by the Heisenberg uncertainty principle, it can be feasible with the current technology as it only requires a tunable two-mode squeezed vacuum (TMSV) source [8] and homodyne detection. We evaluate the information accessible on the signal after the measurements on the cloner modes, assuming their joint as well as independent measurements, and show that optimality of either of the approaches differs depending on whether the modulated signal is below or above the shot noise, defined by the level of vacuum fluctuations. For the sub-shot-noise modulated signals we observe that the cloner is redundant, meaning the independent measurements on its modes are more efficient compared to the joint strategy. In this regime, the standard entangling cloner attack based on the joint treatment of the measurement outcomes from the cloner modes is not optimal anymore and the modes have to be optimally treated independently. On the other hand, for the above-shot-noise modulated signals the cloner is synergistic, when the joint strategy yields more information on the signal. In the regime, when the leakage is minimum, the two approaches are equivalent. Our results are essential for security of practical squeezed-state CV QKD systems against individual attacks as well as reveal nontrivial statistical properties of entangling cloner attacks.

## 2. CV QKD and Entangling Cloner

We consider the generalized Gaussian CV QKD protocol between the trusted parties Alice (*A*) and Bob (*B*) [5] based on the arbitrary Gaussian quadrature modulation of arbitrarily quadrature-squeezed states belonging to a single mode of electromagnetic radiation. Field quadratures are real and imaginary parts of the annihilation and creation operators of a respective mode of the electromagnetic field, which can be introduced as $\hat{x}={\hat{a}}^{†}+\hat{a}$ and $\hat{p}=i\left({\hat{a}}^{†}-\hat{a}\right)$, from which follows the commutation relation for the quadrature operators, $[\hat{x},\hat{p}]=2i$. Introducing the variance of an observable $\hat{A}$ as $Var\left(\hat{A}\right)=\langle {\hat{A}}^{2}\rangle -\langle \hat{A}\rangle \langle \hat{A}\rangle$, one can obtain the quadrature variance of a vacuum or coherent state of light, being equal to one in the used definition and being referred to as the shot-noise unit (SNU). With no loss of generality assuming that the signal states are squeezed in x-quadrature, we denote their x-quadrature variance by $V_{S}$. The squeezed variance is then $V_{S}\leq 1$, which represents the fact that the squeezed quadrature fluctuations are suppressed below the shot-noise level. Accordingly, the fluctuations of the complementary p-quadrature of a pure squeezed state have the variance $1/V_{S}\geq 1$. The Gaussian modulation is applied by displacing the x-quadrature by value $x_{M}$ and the p-quadrature by value $p_{M}$, both taken from the Gaussian distributions with variances $\sigma_{x}$ and $\sigma_{p}$ respectively. Then the overall variance of the signal entering the channel will be $V_{S}+\sigma_{x}:=V$ in x-quadrature and $1/V_{S}+\sigma_{p}$ in p-quadrature (where $\sigma_{x,p}$ are the modulation variances of the respective quadratures).

The modulated states travel through an untrusted Gaussian channel (being the worst-case assumption in Gaussian CV QKD [7]), typically characterized by transmission η and quadrature excess noise ϵ. The channel parameters then explicitly define the strength of an eavesdropping attack in the channel (i.e., how much signal is lost and how much noise is added) and, together with the state preparation parameters, give the upper bound on the amount of information, which is leaking to the channel. If the channel excess noise ϵ is defined with respect to the channel input, the variance on detection outcomes $X_{B}$ on the channel output, measured by the remote trusted party Bob using a homodyne detector, reads

(1) $$V_{B}=\eta \left(V+\epsilon \right)+1-\eta ,$$

which is the result of coupling the noisy signal with variance V+ϵ to vacuum with the coupling ratio η.

**Figure 1:**
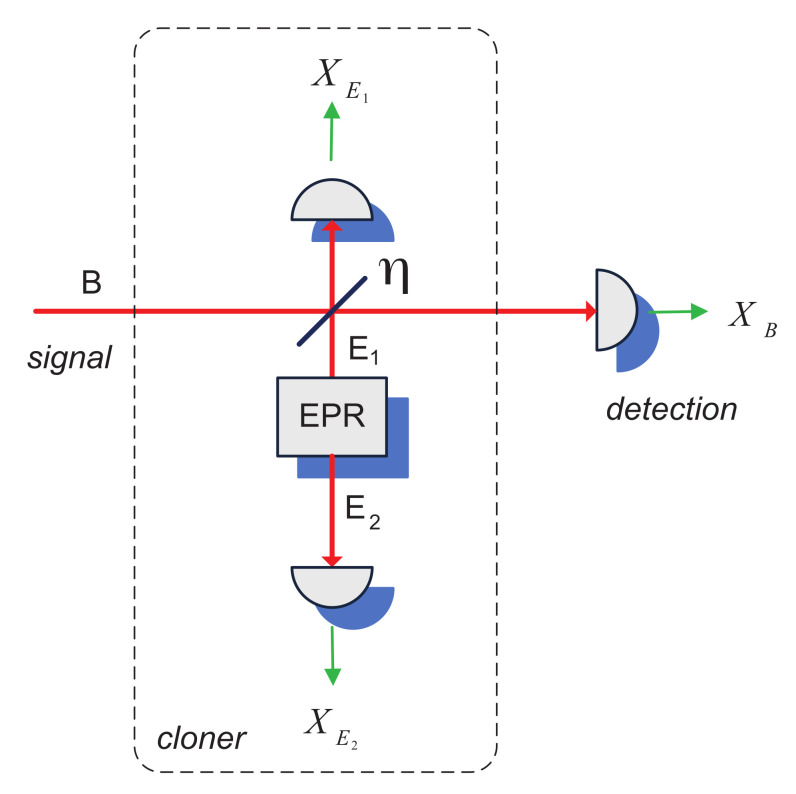
Entangling cloner eavesdropping attack, performed on the continuous-variable quantum communication in a noisy channel with transmittance η.

The optimal individual attack on the Gaussian CV QKD is the entangling cloner attack, which allows an eavesdropper to achieve the bound on the information about the key set by the Heisenberg uncertainty principle [9]. The entangling cloner is a TMSV state with variance *N*; one mode ($E_{1}$) of the cloner is coupled to the signal mode, as shown in Figure 1, while another mode ($E_{2}$) is left intact. Both modes are then measured by an eavesdropper using homodyne detectors, resulting in outcomes $X_{E_{1}}$ and $X_{E_{2}}$, which allows to minimize the uncertainty on the noise added to the signal by mode $E_{1}$, while the signal is measured by the remote party. Entangling cloner can therefore be seen as a purification of a thermal noise in mode $E_{1}$, coupled to the signal, or as a controllable noise addition to the signal after the measurement on mode $E_{2}$, thanks to the strong correlation between the two modes of the TMSV state (see more on security proofs and security analysis methods in CV QKD in the reviews [10,11]).

It is known [9], that in order to mimic the actual channel parameters, the coupling η between the signal and the cloner mode has to be set to the actual channel transmittance, while the variance of the cloner modes has to be N=1+ηϵ/(1−η). Then after the interaction between the signal and mode $E_{1}$, the variance of the data measured by Bob, $V_{B}=\eta V+\left(1-\eta \right)N$, is equivalent to (1), corresponding to the given channel.

To evaluate the upper bound on the leaking information after the entangling cloner attack, we first derive the covariance matrix of the x-quadrature data $X_{B}$, $X_{E_{1}}$ and $X_{E_{2}}$, measured on the signal mode *B* and the cloner modes $E_{1}$ and $E_{2}$, respectively, by the homodyne detectors. The elements of an x-quadrature covariance matrix are obtained as $\gamma_{ij}=\langle \hat{x_{i}}\hat{x_{j}}\rangle -\langle \hat{x_{i}}\rangle \langle \hat{x_{j}}\rangle$, for i=j giving the quadrature variance of a given mode, and for i≠j giving the quadrature covariance (correlation) between the modes *i* and *j*. Such x-quadrature covariance matrix of an entangling cloner in modes $E_{1},E_{2}$ with mode variance *N* prior to interaction with the signal mode *B* reads

(2) $$\gamma_{E_{1}E_{2}}^{\left(x\right)}=\left(\begin{array}{cc} N & \sqrt{N^{2}-1}\\ \sqrt{N^{2}-1} & N \end{array}\right),$$

with *p*-quadrature matrix being the same up to the sign flip in the correlation (off-diagonal) term, corresponding to strong anti-correlation in *p*-quadrature. Coupling η between the mode *B*, containing the modulated signal with variance *V*, and the noise mode $E_{1}$ of TMSV with variance *N*, can be modelled as a beamsplitter interaction, described by the input-output relation for quantum operators in the respective modes as

(3) $${\left(\begin{array}{c} {\hat{a}}_{B}\\ {\hat{a}}_{E_{1}} \end{array}\right)}_{out}=\left(\begin{array}{cc} \sqrt{\eta } & \sqrt{1-\eta }\\ -\sqrt{1-\eta } & \sqrt{\eta } \end{array}\right){\left(\begin{array}{c} {\hat{a}}_{B}\\ {\hat{a}}_{E_{1}} \end{array}\right)}_{in},$$

where η represents the transmittance and 1−η represents the reflectance of a beamsplitter, similarly for the conjugate operators. The resulting covariance matrix then reads

(4) $$\gamma_{BE_{1}E_{2}}^{\left(x\right)}=\left(\begin{array}{ccc} \eta V+(1-\eta )N & \sqrt{\eta (1-\eta )}\left(N-V\right) & \sqrt{\left(1-\eta \right)\left(N^{2}-1\right)}\\ \sqrt{\eta (1-\eta )}\left(N-V\right) & \eta N+(1-\eta )V & \sqrt{\eta (N^{2}-1)}\\ \sqrt{\left(1-\eta \right)\left(N^{2}-1\right)} & \sqrt{\eta (N^{2}-1)} & N \end{array}\right),$$

which, in particular, reflects the fact that the mode $E_{2}$ with variance *N* remains intact (does not interact with the signal), while the initial TMSV correlation between the cloner modes $\sqrt{N^{2}-1}$ is scaled by $\sqrt{\eta }$.

Considering the individual attacks, we analytically evaluate the accessible information in terms of the Shannon (classical) mutual information, defined for a pair of random variables X,Y as I(X:Y)=H(X)+H(Y)−H(X,Y) through the entropies of the form H(X) and the joint entropy of the form H(X,Y). For the Gaussian-distributed continuous variables *X* and *Y*, the mutual information can be expressed as $I\left(X:Y\right)=\left(1/2\right)\log_{2}\left(V_{X}/V_{X|Y}\right)$ (with no loss of generality assuming binary coding), where $V_{X}$ is the variance of variable *X* and $V_{X|Y}=V_{X}-C_{XY}^{2}/V_{Y}$ is the conditional variance expressed through the variance $V_{Y}$ of variable *Y* and the correlation (covariance) $C_{XY}$ between the variables *X* and *Y*. We will also use an extension of the Shannon mutual information to the joint distribution of variables *Y* and *Z* in the form $I\left(X:Y,Z\right)=\left(1/2\right)\log_{2}\left(V_{X}/V_{X|Y,Z}\right)$, where $V_{X|Y,Z}$ is the variance of *X* conditioned on *Y* and *Z*.

In the reverse reconciliation scenario (which is typically considered in CV QKD as it is much more robust against channel losses [7], when the receiver (Bob) is the reference side of the protocol, the information accessible after a joint measurement on the cloner modes can be directly obtained from (4) as

(5) $$I\left(B:E_{1},E_{2}\right)=\frac{1}{2}\log_{2}\left[\eta V+(1-\eta )N\right]\left[\frac{\eta }{V}+\left(1-\eta \right)N\right],$$

which is larger, than the information obtained from the measurement of only the mode $E_{1}$:

(6) $$I\left(B:E_{1}\right)=\frac{1}{2}\log_{2}\left[\eta V+(1-\eta )N\right]\left[\frac{\eta }{V}+\frac{\left(1-\eta \right)}{N}\right],$$

concerned with the replacement of N→1/N in the second term, which corresponds to replacement of $V_{B|E_{1}}\to V_{B|E_{1},E_{2}}$ after the measurement on the mode $E_{2}$ and represents the information advantage of the entangling cloner. Note that the information between the signal and the auxiliary mode $E_{2}$ of the cloner, which reads

(7) $$I\left(B:E_{2}\right)=\frac{1}{2}\log_{2}\frac{N[(1-\eta )N+\eta V]}{\eta NV+1-\eta },$$

is also lower than (5) for the physically valid parameters V>0,N≥1,η∈[0,1].

When the channel noise is absent, i.e., ϵ=0, it means N=1 and the cloner is reduced to two uncorrelated vacuum modes, hence corresponding to the pure channel loss, in which case the measurement on $E_{2}$ has no effect on the information obtained from the measurement on $E_{1}$.

## 3. Minimization of Information Leakage

The condition for minimizing information leakage was obtained in [5] for the case of collective attacks, here we analytically derive this condition in the case of the individual ones. It can be directly obtained from the information leakage to the entangling cloner (5) by taking its derivative by *V*, which reads

(8) $$\frac{dI(B:E_{1},E_{2})}{dV}=\frac{\left(1-\eta \right)\eta N\left(V^{2}-1\right)}{V[\eta V+(1-\eta )N][NV(1-\eta )+\eta ]}.$$

As $I(B:E_{1},E_{2})$ is the convex function of *V* in the physically valid region V∈(0,∞), the minimum is reached when either η=1 (channel is perfect), or V=1, meaning $\sigma_{x}=1-V_{S}$. Therefore, in order to reach the minimum information leakage, the controllable quadrature modulation of the squeezed states, applied by the amplitude or phase modulator (for the amplitude or phase quadrature squeezed state respectively), has to be set in such a way, that the resulting modulated state has the shot-noise variance in the modulated squeezed quadrature. In the case of the noiseless channel ϵ=0 this means that the outputs of the beamsplitter, simulating the channel attenuation, are completely uncorrelated and the information leakage is fully removed [6]. For the noisy channels ϵ≠0 the residual correlations remain due to the noise and the minimum information leakage remains non-zero.

In the regime of the modulated state variance equal to a shot noise, i.e., V=1, we obtain that the mutual information (5) reads

(9) $$I\left(B:E_{1},E_{2}\right){|}_{V=1}=\log_{2}[(1-\eta )N+\eta ].$$

It is straightforward to see by putting V=1 to (6), (7) and comparing to (9) that, in the regime of the minimum leakage, the information between the signal and the jointly measured cloner modes is equal to the sum of the mutual information quantities between the signal and each of the cloner modes:

(10) $$I\left(B:E_{1},E_{2}\right)=I\left(B:E_{1}\right)+I\left(B:E_{2}\right).$$

From this, using the definition of Shannon conditional mutual information I(X:Y|Z)=H(X|Z)+H(Y|Z)−H(X,Y|Z) through the conditional entropies of the form H(X|Z) and the conditional joint entropies of the form H(X,Y|Z), we obtain, that in the regime of minimum leakage

(11) $$I\left(E_{1}:E_{2}\right)=I\left(E_{1}:E_{2}|B\right),$$

where $I(E_{1}:E_{2}|B)$ is the conditional mutual information between the modes $E_{1},E_{2}$, conditioned by the measurement results at *B*, i.e., in the regime of the minimum leakage the conditioning on the residual signal does not change the mutual information between the two cloner modes.

Indeed, we obtain the mutual information $I(E_{1}:E_{2})$ between the modes of the entangling cloner from the elements of the $E_{1},E_{2}$ submatrix of the matrix (4) as follows:

(12) $$I\left(E_{1}:E_{2}\right)=\frac{1}{2}\log_{2}\frac{N[\eta N+(1-\eta )V]}{(1-\eta )NV+\eta }.$$

The conditional mutual information $I(E_{1}:E_{2}|B)$ can be obtained from the conditional covariance matrix $\gamma_{E_{1}E_{2}|B}$, containing variances of the form $V_{E_{i}|B}=V_{E_{i}}-C_{E_{i}B}^{2}/V_{B}$, i={1,2}, and the conditional correlation of the form

(13) $$C_{E_{1}E_{2}|B}=C_{E_{1}E_{2}}-\frac{C_{E_{1}B}C_{E_{2}B}}{V_{B}},$$

which contains the correlations $C_{E_{1}B}$ and $C_{E_{2}B}$ between either of the cloner modes and the signal mode *B*.

We then obtain the resulting matrix as

(14) $$\gamma_{E_{1}E_{2}|B}^{\left(x\right)}=\frac{1}{\eta V+(1-\eta )N}\left(\begin{array}{cc} NV & V\sqrt{\eta (N^{2}-1)}\\ V\sqrt{\eta (N^{2}-1)} & \eta NV+1-\eta \end{array}\right)$$

and obtain the mutual information between the cloner modes conditioned on the signal mode *B* as follows:

(15) $$I\left(E_{1}:E_{2}|B\right)=\frac{1}{2}\log_{2}\frac{N[\eta NV+1-\eta ]}{\eta V+(1-\eta )N},$$

which is evidently the same as $I(E_{1}:E_{2})$ given by (12) when V=1.

In the next section we show the regimes of redundancy and synergy of the entangling cloner, when the equalities (10), (11) do not hold.

## 4. Redundancy and Synergy of an Entangling Cloner

Outside of the optimal regime of the minimum leakage from a Gaussian CV quantum channel, obtained in the previous Section, i.e., when V≠1, one of the quantities in (10) and (11) exceeds another. Similar effect was previously discussed in neuro-science [12] and information theory [13,14]. In particular, the situation when $I\left(B:E_{1},E_{2}\right)>I\left(B:E_{1}\right)+I\left(B:E_{2}\right)$ is referred to as synergy, when jointly systems $E_{1},E_{2}$ provide more information on *B* than separately. Alternatively, when the joint information is less than the sum of the individual ones, i.e., $I\left(B:E_{1},E_{2}\right)<I\left(B:E_{1}\right)+I\left(B:E_{2}\right)$, the system $E_{1},E_{2}$ is called redundant in accessing the information on the system *B*. In this terms, the optimal regime of the minimized leakage $V_{S}=1$ is achieved when entangling cloner $E_{1},E_{2}$ is neither synergistic, nor redundant, while varying the modulated signal variance *V* we access both redundancy and synergy regimes of an entangling cloner, used to optimally estimate the signal.

Importantly, this means that for the sub-shot-noise modulated signal V<1 the optimality of the entangling cloner individual attack [9], well-known in CV QKD and broadly used to study the security of the protocols, does not hold anymore. So do the security bounds set by the Heisenberg uncertainty principle, as the product of uncertainties of $X_{B}$ knowing the outcomes of the measurements on the purifying system *E* and the modulation $X_{A}$ is below 1 SNU. It turns out, that in this regime a more efficient attack can be implemented by treating the measurement outcomes of the two modes of an entangling cloner separately.

**Figure 2:**
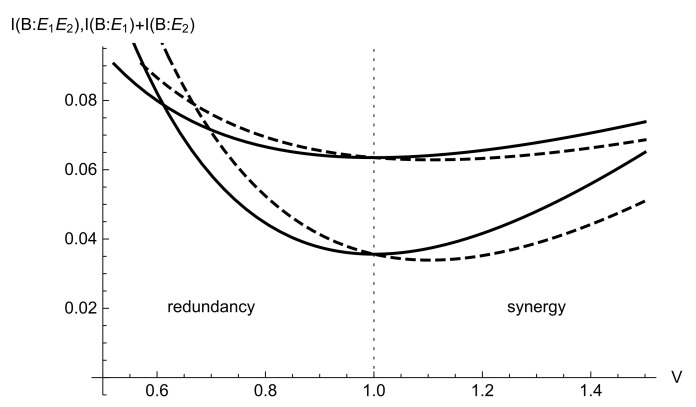
Joint mutual information $I(B:E_{1},E_{2})$ (solid lines) and sum of individual mutual information quantities $I\left(B:E_{1}\right)+I\left(B:E_{2}\right)$ (dashed lines) between the cloner modes and the signal versus signal variance *V* at channel transmittance η=0.1 (upper plots) and η=0.5 (lower plots) and cloner variance N=1.05 SNU (equivalent to the channel noise ϵ=0.45 SNU at η=0.1 and ϵ=0.05 SNU at η=0.5).

The typical regimes of an entangling cloner inferring a transmitted signal are given in Figure 2 in terms of two types of mutual information analytically given by (5)–(7) with respect to the signal variance for different values of channel transmittance (cloner coupling ratio) η. It is evident from the graph that the joint information $I(B:E_{1},E_{2})$ is constantly minimized upon signal variance being equal to a shot-noise unit, V=1. When the signal remains squeezed (V<1) the redundancy of the cloner is observed, while as signal becomes more noisy than the shot noise, V>1, the synergy of the cloner takes place.

**Figure 3:**
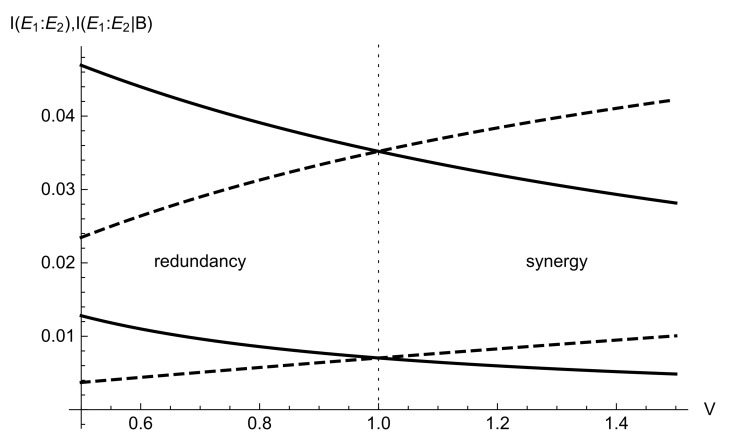
Mutual information between the cloner modes before $I(E_{1}:E_{2})$ (solid lines) and after $I(E_{1}:E_{2}|B)$ (dashed lines) conditioning on the measurements on mode *B* versus signal variance *V* at channel transmittance η=0.1 (lower plots) and η=0.5 (upper plots) and cloner variance N=1.05 SNU.

Similarly, we can revert the scheme and consider the communication between the cloner modes $E_{1},E_{2}$ and the role the measurement results on the mode *B* take in this communication. Out of the minimum leakage regime V=1 given by (11), when the state in mode *B* before the coupling η has shot-noise variance in the measured quadrature, the mutual information between the cloner modes can be increased on decreased by conditioning on the measurement results at *B*. The typical dependencies of $I(E_{1}:E_{2})$ and $I(E_{1}:E_{2}|B)$, given by (12) and (15), i.e., before and after conditioning on *B*, respectively, depending on the variance of the state in mode *B* prior to the interaction with the cloner, are given in Figure 3.

It is evident from the plots in Figure 3, that, contrary to the mutual information quantities between the channel output and the cloner modes (joint or separate) given in Figure 2, which are the convex functions of the modulated signal variance *V*, the mutual information between the cloner modes $E_{1},E_{2}$ continuously increases with the increase of the signal variance *V* if conditioned on the output of mode *B* or continuously decreases without the conditioning. Indeed, the variance of mode *B* then plays the role of an external noise, which either contributes to the mutual information once conditioning is performed, or does not, once the measurements on *B* are not taken into account. We further extend the scheme to interaction between two entangling cloners and show how conditioning on an auxiliary cloner modes changes the mutual information between the modes of the main one.

## 5. Two Interacting Entangling Cloners

**Figure 4:**
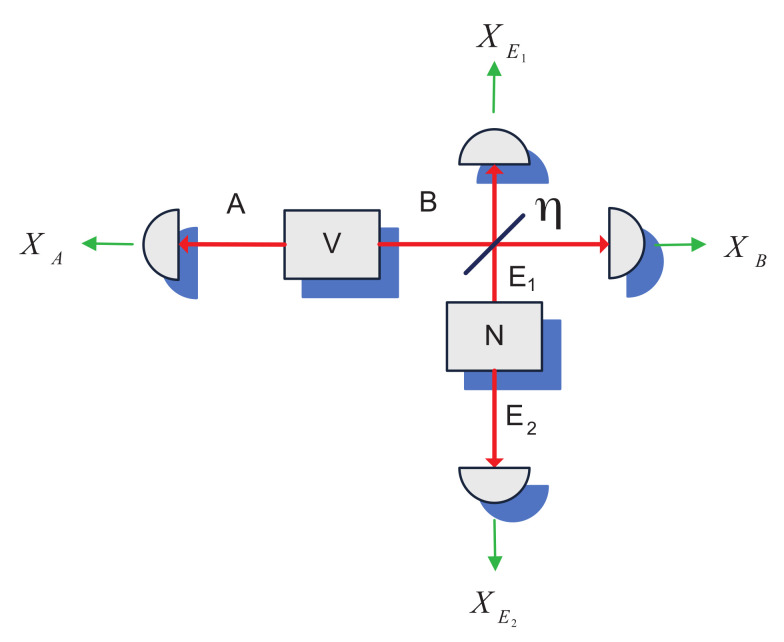
Two entangling cloners: one with variance *V* in modes A,B and another with variance *N* in modes $E_{1},E_{2}$, interacting between modes *B* and $E_{1}$ with coupling η.

To generalize the result of the effect of conditioning on the external noise in mode *B* on the mutual information between the modes of an entangling cloner, we consider the scheme of two mutually interacting entangling cloners, as shown in Figure 4.

Prior to interaction each of the cloners can be described in x-quadrature by a covariance matrix of the form (2) with variances *N* and *V*. We consider the communication between the cloner modes $E_{1}$ and $E_{2}$ (which can be also seen as a purification of the modulation performed on a single mode $E_{1}$ [9]) and study the effect of conditioning on (using the knowledge of the quadrature values of) the auxiliary cloner modes A,B. We analytically derive the covariance matrices of the two cloners and obtain the respective mutual information quantities as discussed below.

After the coupling η between the modes *B* and $E_{1}$ the x-quadrature covariance matrices of the two cloners have the form

(16) $$\gamma_{AB}^{\left(x\right)}=\left(\begin{array}{cc} V & \sqrt{\eta (V^{2}-1)}\\ \sqrt{\eta (V^{2}-1)} & \eta V+(1-\eta )N \end{array}\right),$$

(17) $$\gamma_{E_{1}E_{2}}^{\left(x\right)}=\left(\begin{array}{cc} \eta N+(1-\eta )V & \sqrt{\eta (N^{2}-1)}\\ \sqrt{\eta (N^{2}-1)} & N \end{array}\right),$$

and their correlation matrix reads

(18) $$\sigma_{ABE_{1}E_{2}}^{\left(x\right)}=\left(\begin{array}{cc} -\sqrt{\left(1-\eta \right)\left(V^{2}-1\right)} & \sqrt{\eta (1-\eta )}\left(N-V\right)\\ 0 & \sqrt{\left(1-\eta \right)\left(N^{2}-1\right)} \end{array}\right).$$

Without the conditioning, the mutual information $I(E_{1}:E_{2})$ between the cloner modes is given by (12). After the conditioning performed on mode *B*, the x-quadrature covariance matrix of the state in modes $E_{1},E_{2}$ has the form (14) and the respective conditional mutual information $I(E_{1}:E_{2}|B)$ is given by (15). As the variance *V* of the cloner in modes A,B is always V≥1 (equality means the cloner reduces to two uncorrelated vacuum states), which is implied by the physicality constraint given by the Heisenberg uncertainty principle [15], the mutual relation of those two mutual information quantities for the two-cloner scheme corresponds to the right part of the plot in Figure 3. Hence, conditioning on the state in mode *B* having variance larger than the shot noise level improves the mutual information between the cloner modes. Since the thermal state in mode *B*, after it is split between modes *B* and $E_{1}$ by the beamsplitter η, introduces correlations between the two modes, the conditioning can be seen as application of additional controllable modulation to the mode $E_{2}$ of the entangling cloner. Such additional modulation is known to improve the entangled resource for quantum communication [16].

If instead of measurement and conditioning on mode *B*, the measurement on mode *A* is taken into account, the covariance matrix of the conditional state in modes $E_{1},E_{2}$ obtains the form (note that the mode $E_{2}$ is not affected as it is not correlated to mode *A*):

(19) $$\gamma_{E_{1}E_{2}|A}^{\left(x\right)}=\left(\begin{array}{cc} \frac{\eta NV+1-\eta }{V} & \sqrt{\eta (N^{2}-1)}\\ \sqrt{\eta (N^{2}-1)} & N \end{array}\right),$$

the resulting mutual information $I(E_{1}:E_{2}|B)$ is then exactly the same as $I(E_{1}:E_{2}|A)$ given by (15). Hence, conditioning on either mode of the auxiliary cloner A,B improves the mutual information between the modes $E_{1},E_{2}$ due to the strong correlation between the modes *A* and *B*. Finally, if the measurements on both modes A,B is taken into account, the resulting conditional matrix reads

(20) $$\gamma_{E_{1}E_{2}|AB}^{\left(x\right)}=\frac{1}{\eta +(1-\eta )NV}\left(\begin{array}{cc} N & \sqrt{\eta (N^{2}-1)}\\ \sqrt{\eta (N^{2}-1)} & \eta N+(1-\eta )V \end{array}\right),$$

and the resulting mutual information $I(E_{1}:E_{2}|AB)$ is exactly the same as $I(E_{1}:E_{2})$ without any conditioning, given by (12). Hence, conditioning on both modes of the auxiliary cloner cancels the positive effect of the additional correlations in modes *B* and $E_{1}$.

## 6. Discussion

Inspired by the optimality of entangling cloner as an individual attack in Gaussian CV QKD, we have analyzed the mutual information quantities between a modulated signal and an entangling cloner. We have derived the condition for minimization of information leakage under the individual attacks, which is the same as for the collective ones. We have then shown, that when the information leakage from the Gaussian channel is minimized, the joint measurement on the entangling cloner modes yields the same mutual information as when the measurement data is taken independently. In this regime of minimum leakage, the mutual information between the modes of a cloner does not change with conditioning on the residual signal. Out of this regime the cloner is either redundant, when the signal is moduated below the shot noise, which means that obtaining the information on the signal from the joint measurement on the cloner modes is less effective than from the sum of individual information quantities, or synergistic, which is the typical known regime for the entangling cloner, when the signal is modulated above the shot noise and treating the measurement data from the cloner modes jointly is more efficient. Importantly, our result shows that entangling cloner with joint measurement on the cloner modes is not an optimal individual attack for the signals modulated below the shot noise. This affects the whole security analysis of CV QKD with the sub-shot-noise-modulated signals in the assumption of individual attacks, particularly in the case of low modulation regime, which can be used to compensate for the low error correction efficiency [5] (e.g., due to high-speed real-time processing of the key data). Despite the fact, that individual attacks are less efficient than collective ones in CV QKD, this class of attacks is important as the security analysis can be reduced to the individual attacks, e.g., in the free-space and satellite CV QKD, where visibly controllable line of sight suggests the absence of bulky equipment capable of collective attacks, such as quantum memories, which can improve the protocol applicability [17]. Furthermore, we extended our consideration to the scheme with two interacting entangling cloners and shown, that while an auxiliary cloner acts as an external noise, degrading the mutual information between the main cloner modes, the conditioning on either of the modes of the auxiliary cloner improves the mutual information of the main cloner, providing additional correlations to the entangled state [16]. The effect vanishes, when the conditioning is taken on both the modes of an auxiliary cloner. Note, that we consider interaction between two modes, one of each cloner, while if both modes of each cloner are interacting, this can be seen as a correlated cross talk and can be effectively removed for entanglement distribution [18] and QKD [19].

## Data Availability

Not applicable.

## Abbreviations

The following abbreviations are used in this manuscript:

CV	Continuous-variable
QKD	Quantum key distribution
SNU	Shot-noise unit
TMSV	Two-mode squeezed vacuum
